# What can we do when the smoke rolls in? An exploratory qualitative analysis of the impacts of rural wildfire smoke on mental health and wellbeing, and opportunities for adaptation

**DOI:** 10.1186/s12889-021-12411-2

**Published:** 2022-01-06

**Authors:** Anna Humphreys, Elizabeth G. Walker, Gregory N. Bratman, Nicole A. Errett

**Affiliations:** 1 Department of Health Systems and Population Health, University of Washington, Box 351621, 3980 15th Ave NE, Fourth Floor, Seattle, WA 98195 USA; 2 Department of Environmental and Occupational Health Sciences, University of Washington, 4225 Roosevelt Way NE, Suite 100, Seattle, WA 98105 USA; 3 Clean Air Methow, Methow Valley Citizens Council, PO Box 774, Twisp, WA 98856 USA; 4 School of Environmental and Forest Sciences, University of Washington, Anderson Hall, Box 352100, Seattle, WA 98195 USA

**Keywords:** Wildfire smoke, Wellbeing, Qualitative methods

## Abstract

**Background:**

Extreme, prolonged wildfire smoke (WFS) events are becoming increasingly frequent phenomena across the Western United States. Rural communities, dependent on contributions of nature to people’s quality of life, are particularly hard hit. While prior research has explored the physical health impacts of WFS exposure, little work has been done to assess WFS impacts on mental health and wellbeing, or potential adaptation solutions.

**Methods:**

Using qualitative methods, we explore the mental health and wellbeing impacts experienced by community members in a rural Washington State community that has been particularly hard hit by WFS in recent years, as well as individual, family, and community adaptation solutions. We conducted focus groups with residents and key informant interviews with local health and social service providers.

**Results:**

Participants identified a variety of negative mental health and wellbeing impacts of WFS events, including heightened anxiety, depression, isolation, and a lack of motivation, as well as physical health impacts (e.g., respiratory issues and lack of exercise). Both positive and negative economic and social impacts, as well as temporary or permanent relocation impacts, were also described. The impacts were not equitably distributed; differential experiences based on income level, outdoor occupations, age (child or elderly), preexisting health conditions, housing status, and social isolation were described as making some residents more vulnerable to WFS-induced physical and mental health and wellbeing challenges than others. Proposed solutions included stress reduction (e.g., meditation and relaxation lessons), increased distribution of air filters, development of community clean air spaces, enhancing community response capacity, hosting social gatherings, increasing education, expanding and coordination risk communications, and identifying opportunities for volunteering. Findings were incorporated into a pamphlet for community distribution. We present a template version herein for adaptation and use in other communities.

**Conclusions:**

Wildfire smoke events present significant mental health and wellbeing impacts for rural communities. Community-led solutions that promote stress reduction, physical protection, and community cohesion have the opportunity to bolster resilience amid this growing public health crisis.

**Supplementary Information:**

The online version contains supplementary material available at 10.1186/s12889-021-12411-2.

## Background

Anthropogenic climate change is increasing the risk for devastating wildfires [1]. Over the past thirty years, the area burned by forest fires in the western U.S. has nearly doubled due to drier forests [2]. About half of the observed increase in length of fire season and days with high fire risk has been attributed to climate change [2]. Warmer temperatures also generate more lightning, the main natural cause of fires [2].

Wildfires can produce an enormous amount of smoke, containing fine particulate matter (PM2.5-PM10), carbon monoxide, nitrous oxide, methane, volatile organic compounds, and other air toxins known to be harmful to human health [2] that can travel thousands of miles downwind [3]. Projected increases in wildfire events may result in an additional 25 million people exposed to multi-day wildfire smoke events in the Western United States by the middle of the twenty-first century [4].

Even when wildfire is not a direct threat to life, associated wildfire smoke (WFS) events take physical, psychological, and economic tolls on residents [2, 5]. Research to date has primarily explored its physical health effects, and has identified associations between WFS and respiratory morbidity, cardiovascular health impacts, and all-cause mortality [2]. Research has also found strong positive associations of WFS exposure with exacerbated COPD, asthma, pneumonia, and bronchitis [2, 6]. The effects may be worse for fetuses, children, pregnant women, people with respiratory disease, African American people, people with lower socio-economic status and older adults, particularly women [2].

Yet only a small number of studies have explored the impacts of extreme WFS events on mental health and wellbeing. Research on impacts of multi-day wildfire smoke exposure on wellbeing and mental and behavioral health is necessary to complement related studies that have documented the negative effects of air pollution more broadly on self-reported distress [7]. However, given that extreme, persistent WFS events have historically had the largest impacts on rural communities, wellbeing impacts may be challenging to detect through retrospective epidemiologic analyses given low population density, low healthcare-seeking behavior, and regionalization of specialized healthcare services. In a major review of the literature of health outcomes from wildfire smoke exposure by Reid et al., four of six studies focused on mental health that matched inclusion criteria were determined to have higher bias potential [6]. In the two remaining studies, one found no increase in mental health hospitalizations during a 1987 California smoke event and the other found no increase in mental illness-related physician visits during the 2003 wildfire season in British Columbia [6]. A small number of qualitative studies have begun to explore wellbeing impacts experienced following event-specific WFS exposure. For example, Dodd et al. studied the mental health effects of prolonged WFS exposure during a record-breaking smoke event in a northern Canadian rural population [8]. Interview participants reported elevated feelings of depression, irritability, fear, hopelessness, anxiety, isolation, and lethargy [8].

Rural communities may be particularly at risk for mental health and wellbeing impacts of WFS exposure. Across the arid rural West, where much of the wildfire risk is high, economies often depend on the outdoors in the summer, with industries including tourism, agriculture, and construction. Many residents have chosen to live in these areas due to the close proximity and access to natural resources and the natural environment, with ongoing access to outdoors being crucial for wellness [9, 10].

Concomitantly, rural communities may be less prepared to deal with mental health and wellbeing impacts of WFS. Rural communities generally face health provider shortages due to economic, geographic, or social factors [11]. Many rural communities in America face elevated rates of mental health challenges among their residents, with limited access to behavioral health providers [11, 12]. Rural communities experience a disproportionate lack of physical and mental health integration and stigma of mental healthcare as further barriers [13]. Additionally, counselors may not be trained in interactions specific to rural health settings, including professional isolation and dual relationships between counselors and patients [13, 14].

## Methods

### Study aims

This study sought to describe how extreme, persistent smoke events impact mental health and wellbeing and how community members have coped with these impacts and identify individual and community-level adaptation opportunities to mitigate wellbeing impacts in future wildfire events.

### Conceptual framework

While mental illness entails an occurrence of a clinically defined cognitive, affective, and behavioral disorder, mental health and wellbeing includes multiple cognitive and affective aspects, such as happiness, self-actualization, resilience, and healthy relationships [10]. Our study leverages an operational definition of wellbeing based in strengths-based and positive psychology: “The balance point between an individual’s resource pool and the challenges faced,” visually represented as a see-saw balancing psychological, social, and physical resources and challenges [15]. According to Dodge et al.’s multi-disciplinary review of research on wellbeing, “stable wellbeing is when individuals have the psychological, social and physical resources they need to meet a particular psychological, social and/or physical challenge” [15]. In relationship to challenges, wellbeing levels operate along inverted “u-shape:” wellbeing declines when challenges exceed resources, peaks when challenges are met with adequate resources, and decreases when resources exceed challenges [15]. This model of wellbeing proposes that humans need challenges to avoid stagnation and implies that challenges are not to be avoided but are to be met with increased resources.

### Study setting

We situated our work in the Methow Valley (MV), a community in Okanogan County, Washington, United States of America. Given its natural beauty and proximity to outdoor recreational opportunities and the North Cascades National Park, the MV is a vacation destination for many and a home to about 6000 people [16]. About half of MV residents live in the towns of Winthrop and Twisp and the unincorporated communities of Mazama and Carlton, while the remainder dwell in outlying areas spread along a 60-mile watershed. According to 2019 American Community Survey 5-year estimates, the MV has a predominantly white population (95%), a household median income of $57,500, and a poverty rate of 12.5% [16].

The MV’s geophysical characteristics, reliance on wood stoves for home heating, outdoor burning for organics disposal, and proximity to prescribed burning necessary for wildfire mitigation and forest health have caused the area to experience some of the worst PM2.5 air pollution in the state, in addition to its experience with extreme, persistent WFS events for 6 of the past 9 summers (2012, 2014, 2015, 2017, 2018, 2020) [17]. In response, Clean Air Methow (formerly Methow Valley Clean Air Project) was established in 2013 to lead community-based, year-round programming to improve air quality where possible, e.g., facilitating woodstove exchanges, conducting chipping events as an alternative to outdoor burning, conducting outreach on wildfire smoke preparedness, coordinating workshops on clean home heating, and maintaining one of the largest rural networks of low-cost air sensors (PurpleAir®) in the world as part of their Clean Air Ambassador program [18, 19]. Projects are prioritized in response to community questions and needs with a strong emphasis on ensuring access to clean air for at-risk populations.

Since 2016, Clean Air Methow has worked with members of the University of Washington’s (now) Collaborative on Extreme Event Resilience and Interdisciplinary Center for Exposures, Diseases, Genomics and the Environment on community-engaged research related to wildfire smoke and health. Through its ongoing engagement with the community, including through its role in the development of the MV’s Climate Action Plan, Clean Air Methow has identified wellbeing impacts of WFS as a top community concern. This responsive, collaborative research seeks to respond to community-identified information needs regarding experienced mental health and wellbeing impacts of WFS events and solutions to mitigate such impacts.

#### Study design

Focus groups were used to collect data from community members, and key informant interviews were used to collect data from health and social service providers.

### Data collection

#### Focus groups

In November 2019, three 90-min focus groups with community members were conducted at a community center in Twisp, WA, one of the larger towns in the MV. Focus group objectives were to understand (1) how persistent, extreme WFS events have impacted participants’ mental health and wellbeing, (2) how they have coped, and (3) what they perceived as opportunities for supporting WFS event-related mental health and wellbeing.

Focus group facilitators were recruited from Clean Air Methow and local health and social service provider network. Facilitators were provided with a recorded PowerPoint training video, a briefing document and a facilitation guide in written form (Additional File 1). A researcher (AH) spoke with each facilitator over the phone to ensure they felt prepared and to answer any questions they had and reviewed the training materials with the facilitators during the hour before the community event began.

Because the MV is a small community, facilitators may have had preexisting relationships with focus group participants. Prior to the start of the session, facilitators discussed their potential bias or assumptions, as well as their personal and/or professional goals and interests related to the research. Facilitators also had the opportunity to share their personal and/or professional goals and interests related to the research with focus group participants during a 30-min panel at the conclusion of the focus groups. Participation in focus groups was open to any full or part-time resident of the MV at least 18 years of age and that was a fluent English speaker. A total of 13 individuals participated in the three focus groups.

Clean Air Methow recruited participants through their networks (e.g., the Clean Air Ambassadors Program and health service providers), social media outreach, word of mouth, and fliers posted in diverse locations (e.g., the community center, library, grocery stores, healthcare providers, and social service providers). Focus group participants received free childcare if needed, a pizza dinner, refreshments, and a $10 gift card to the local supermarket. While recruitment sought diverse representation in terms of socioeconomic status, medical vulnerabilities to wildfire smoke, and racial/ethnic groups, focus group participants were ultimately a convenience sample.

Facilitators recorded interviews using digital audio recorders and took real-time notes on large sticky pads to allow participants to confirm accuracy of interpretation of their contributions and allow for the generation of feedback and ideas. Each participant was provided with a pen and paper in case they did not wish to share any answers with the larger group. These notes were transcribed after the event and included in the analysis.

#### Focus group design

Prior to the start of the focus groups, focus group participants completed a demographics form. Participants were randomly assigned to one of the three facilitators. Verbal consent was obtained from all participants at the start of the focus group, and participants and facilitators provided self-introductions (name, length of time participants had lived in the MV, and prior felt impacts of WFS events). The facilitators then used a discussion guide (Additional File 1) to lead a conversation about the impacts of WFS events, including physical, psychological, and social impacts. Next, facilitators led focus group participants in an activity (outlined in the facilitation guide, Additional File 1) to describe individual, community and county-level coping strategies used in the past and ideas for the future.

### Key informant interviews

In December 2019 and January 2020, 16 semi-structured individual interviews were conducted with local health and social service providers (“key informants”) who were identified using a combination of purposeful sampling, a nonprobability sampling technique used to recruit individuals with rich experience or expertise [20], and snowball sampling. Here, the population of experts included professionals in the MV community who provided health or social services to diverse MV community members during and after WFS events. Clean Air Methow provided an initial list of potential key informants that met study inclusion criteria. Key informants also suggested additional participants, who were subsequently assessed against study inclusion criteria. Eligible individuals were then recruited to participate in the study (i.e., snowball sampling).

Potential participants were provided with an email invitation describing the study and asked to respond within 2 weeks. Follow-up invitations were sent, if necessary, 2 weeks later. Key informants had the opportunity to receive a copy of *A Fire Story* by Brian Fies, a graphic novel depicting the author’s experience during the Tubbs fires in California.

Interviews were scheduled based on key informants’ availability, were performed over the phone and lasted 20 min to 1 h. Before the interview began, key informants were read an informed consent statement (Additional File 2) and given the chance to ask any questions. Verbal consent was obtained before the interview began.

Interviews were guided by a semi-structured interview guide (Additional File 2) and explored: (1) challenges to physical, mental, and social wellbeing during WFS events; (2) methods and opportunities for coping; (3) suggestions for a community toolkit, including medium, messenger, and information.

### Data analysis

Interviews and focus group recordings were professionally transcribed. These recordings, along with participant notes and written responses to focus group prompts, were thematically analyzed using a combined inductive and deductive approach, adapted from the Framework Approach to applied qualitative analysis [21, 22]. In the deductive phase, codes were identified based on the research questions and conceptual framework. In the inductive phase, the primary researcher (AH) read and re-read transcripts and took detailed notes to identify additional categories, or themes, that emerged during the interviews and focus groups. Emergent themes that were not already reflected in the deductive coding schema were memorialized into codes and given formal definitions and examples of when to apply. Learnings from this data familiarization process also informed clarifications and contextualization of deductive code definitions, as well as the addition of subcodes. Codes developed through both the deductive and inductive phases were integrated and institutionalized into a codebook. This codebook was then used for line-by-line coding by the primary researcher.

Ten percent of the interviews were double-coded to refine the codes and ensure consistency between coders, with the goal of 75% inter-coder reliability, defined here as the proportion of total code applications ([concordant code applications] + [additional code applications by coder A] + [additional code applications by coder B]) that were concordant (i.e., instances where coder A applied the same codes to the same text as coder B). Fifty-eight percent was achieved during the first round of double-coding, after which several codes were re-worded to be more specific. After these adjustments, another 10 % of interviews were co-coded and 82% inter-rater reliability was achieved.

QSR International Pty Ltd. (2018) NVivo (Version 12) software was used to code the data. A second round of coding occurred to organize the data into more specific subcodes. Then, analytic memos for each code and subcode were developed to summarize and synthesize ideas expressed during the focus groups and key informants, noting similarities and differences between the groups. These synthesized findings were compared to the raw data to confirm that the ideas represented reflected the interview and focus group discussions.

## Results

Focus group participants’ mean age was 46.6, almost all identified as White (92%), and most had a college degree (77%). About half of the participants had a child under age ten (46%). One participant was unemployed, and all others were employed full or part-time. More than half of focus group participants (61%) generated over $80,000 per year in household income. Key informants did not report demographic data, as they were acting in a professional capacity. Professions for key informants included healthcare provider (5), complementary & alternative medicine practitioner (yoga, meditation, somatic therapy) (4), community and social service provider (3), mental health practitioner (2), school nurse (1), and emergency medical technician (1).

Primary concerns around WFS impacts included mental health (primarily anxiety, depression, isolation, and lack of motivation), physical health (primarily respiratory illnesses and loss of exercise), and expenses and loss of work. At the same time, a few participants described how firefighting employment opportunities associated with WFS events brought economic activity to the region. Social impacts were both negative (less community cohesion and gatherings) and positive (shared resilience and support). Factors for being at higher risk for negative effects of WFS included lower income level, outdoor occupations, age (child or elderly), preexisting health conditions (primarily respiratory conditions), pregnancy, housing insecurity/homelessness, and social isolation.

The most prominently expressed adaptation need was a clean air community gathering space, either in a new building or in a preexisting space with excellent air quality. Participants also emphasized the need for community events and activities. Spaces for children to play was a main concern.

Interventions on the individual-level included stress reduction methods and support groups, free air filters and masks for low-income households, and volunteer opportunities. Enhanced communication and education around various aspects of WFS impacts was encouraged.

Below, we present context-rich descriptions of key themes that emerged across key informant and focus groups (herein referred to as “participants”). Impacts and suggested interventions are summarized in Table 1.

**Table 1:** Summary of WFS impacts and suggested interventions

WFS Impacts/ Challenges	Examples of Participant-Suggested Interventions
Anxiety/ Depression	Stress reduction: meditation, relaxation, support groups
Isolation	Volunteer opportunities
	Resource provision
	Mutual aid
Respiratory conditions	Free/low-cost air filters and masks for low-income households
Community dissolution	Gatherings to boost morale and mental wellbeing
	Clean air community space(s)
Lack of awareness of WFS exposure or health impacts	Informational educational campaigns during WFS events
	Providing communications in multiple formats
	Adding push notifications to apps when AQI reaches harmful levels

### Mental health and wellbeing impacts

All participants discussed the mental health and wellbeing implications of WFS events. Anxiety, depression, isolation, and lack of motivation were the most frequently expressed aspects among both groups and are expanded upon below. About a third of key informants identified a lack of available, insured access to mental healthcare; however, focus group participants did not mention this concern.

Participants described lingering psychological effects of WFS events into the winter or the next year, including one focus group participant describing a resurgence of trauma when they drove through fog, and another saying that they were on high alert for signs of WFS during what turned out to be a smokeless summer.

#### Anxiety

Most participants associated anxiety, worry, or stress with WFS events. Key informants noted that individuals who were from marginalized or at-risk groups were more susceptible to anxiety than others. Sources of anxiety included physical health effects, isolation, and worry about children. A key informant who was a healthcare provider noticed that patients would come into clinics for unnecessary medical testing, particularly for respiratory conditions, due to their heightened anxiety, and a mental health provider speculated that the anxiety gets worse as the season continues, peaking around October or November. One participant explained, “*The stress response or the mental health impact of the persistent smoke just can’t be understated. It’s truly significant when you can’t see. You can’t see, breathe, or do what you want to do*.”

#### Depressive symptoms

Many participants discussed experiencing depression, or that their fellow community members experienced depression, during or surrounding WFS events. Participants also described exacerbations of existing depression during WFS events. For example, one key informant said that their patients ask for higher doses of antidepressants during WFS events. Another said that seasonal depression intensifies after a fire season. One participant said, “*I think there was exacerbation of depressive symptoms, because usually they’ll get better in the summer, but people were staying inside, people lived in dark, and it was grim outside*.”

#### Isolation

Most participants discussed social isolation experienced by the MV population during WFS events. Several participants mentioned depression leading to or stemming from social isolation. One key informant described people who were already more vulnerable to mental health challenges as more at risk, explaining, “*It probably isolates those who are already isolated and brings together the people who tend to be more social*.” Several participants also expressed feelings of alienation in a community with pre-existing social connections: “*People tend to gather in their groups when there’s an emergency event like that, and it becomes a little bit cliquey, and I didn’t feel like I could easily connect with people in the emergency situations that. I felt a little bit marginalized. And that probably can happen with single people*.”

#### Lack of motivation

Many participants associated WFS events with feeling “down,” “malaise,” “bleak,” “worn down,” “heavy,” “unmotivated,” or “dampened.” Some speculated that the feeling of “dullness” gets worse toward the end of summer: “*You don’t want to go outside. You’re less motivated, more prone towards depression*.”

### Physical health impacts

All participants discussed physical health consequences of WFS events. Respiratory issues were by far the most discussed across both groups, which is reflected in current research on WFS and health [3, 19]. The main concern expressed was exacerbated asthma, as well as with respiratory infections, difficulty breathing, and exacerbated COPD. Several participants were particularly concerned with the health effects on children. Many participants described WFS-induced health challenges (e.g., pneumonia or lung infections) lasting or appearing past the events themselves and expressed particular concern for the cumulative effects for children and teenagers exposed to WFS. Participants also expressed concern about shallow breathing and sleeplessness.

Loss of physical exercise was an additional chief concern, including for children. Many participants described an outdoors-oriented culture in the MV and discussed that WFS events’ prevention of outdoor activity affects the physical and social wellbeing of MV residents.

### Economic impacts

Most participants identified economic loss as a result of WFS events in the following areas, listed in order of frequency: the tourism industry, restaurants/pubs, cafes, outdoor work, hospitality, theater, and weddings. One KI identified the expense of masks and air purifiers as a financial stressor; another expressed that residents leaving the MV during WFS events hurt the local economy. Conversely, two participants expressed that fire boosts the economy because wildland firefighting provides jobs, and that firefighters spend money in the community.

### Social impacts

Most participants discussed cancelled events preventing community interactions, leading to disruptions to the community’s social fabric. Many mentioned a recreational culture (hiking, running, and biking) being disrupted and as a result seeing their community less frequently during WFS events. Other cancelled events included music festivals, barbeques, swimming, and book clubs.

Most participants attributed WFS to weakening community cohesion. Many expressed that depression, fear, or isolation may make people more reticent to leave their homes. Several participants described social interactions during WFS events revolving around the challenges of the current situation. These participants expressed concern that this resulted in social interactions bringing moods further down, rather than bringing relief.

Conversely, most participants also speculated that WFS events may strengthen community connections due to the experience of shared adversity and the need to work together and help one another. Several participants suggested that preexisting community cohesion and a culture of helpfulness and watching out for one another increase the MV community’s resilience, describing the adversity as a source of strength.

### Temporary or permanent relocation

Most participants brought up local residents leaving or planning to leave during WFS events. Many associated the ability to leave with disproportionate economic or occupational privilege and described people with less resources having no escape. Many participants discussed feelings of guilt or stress about leaving the community during these times. Several expressed a staunch commitment to staying and taking care of each other. Several participants mentioned leaving to escape the smoke, only to end up in another place, such as Alaska, with WFS, and said that they now plan their lives or summers around anticipated WFS events. One participant reported changing jobs to be more flexible in their ability to leave. One key informant said that one of their patients had moved away due to WFS events irritating their asthma. Another speculated that a third smoky summer in a row would drive people to people move away.

### At-risk groups

Most participants reported that while WFS events affect everyone in the area, lower income level, outdoor occupations, age (child or elderly), preexisting health conditions (primarily respiratory conditions), pregnancy, housing insecurity/homelessness, and social isolation make some residents more vulnerable to WFS-induced physical and mental health and wellbeing challenges than others. Many participants noted that recommended interventions such as air conditioning and filtration, as well as other general recommendations, may not be affordable or realistic for everyone.

Participants also expressed concerns about youth as an at-risk population, in part due to the potential for cumulative, long-lasting effects. Other concerns included refusal to wear face masks, increased asthma events, increased screen time, smoke exposure during outdoor sports, lack of exercise, and poor air quality in schools, daycare centers, or friends’ houses.

Many participants identified outdoor workers as at risk of lost wages if they fall ill because of WFS exposure and cannot work, or if hours are cut due to WFS events. Several participants mentioned the urgency of completing construction projects and that the workers and the community both depend on the money generated by outdoor work. A few participants reported that workers may not always wear face masks due to prolonged discomfort.

## Intervention opportunities

### Mental and physical health

**Fig. 1:**
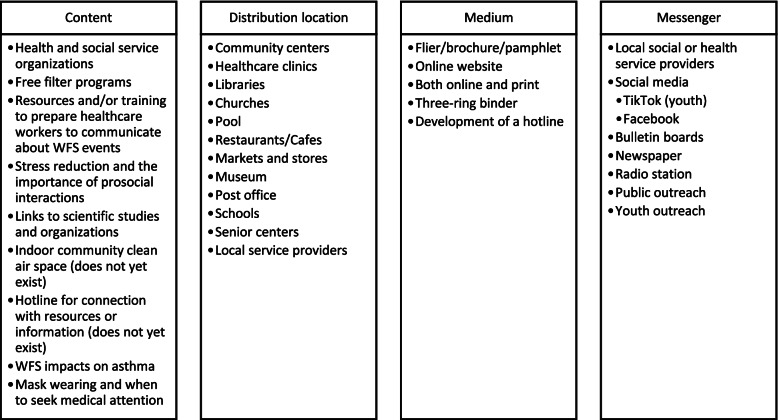
Suggested content, distribution location, medium, and messenger for a wildfire smoke and wellbeing toolkit

Participants identified several interventions that may increase mental and physical health for individuals during WFS events (Fig. 1). Stress reduction was the central theme for mental health and wellbeing, including through meditation and relaxation lessons in a support group. Some participants had already participated in or facilitated these classes and reported that they were successful for reducing stress. Free air filters and masks for low-income households was the chief recommended physical health intervention across both groups, along with education on air filter and box fan filter effectiveness. Pairing indoor air filtration with air quality monitoring was suggested as an intervention to promote mental health and wellbeing to provide a feeling of relief and mitigate helplessness.

### Community clean air space

A community clean air space was overwhelmingly the most common need expressed. Participants discussed that this could provide a space for residents to gather to bolster mental and social wellbeing, and to engender physical activity; some suggested amenities such as a pool, gym, and open space for dance and movement. One participant stated, *“We need a community center. We need somewhere for people to go that has a full-on air filter system. An actual clean air system that’s open to the community … There needs to be a place for community to come together to help bring morale up during that time and it needs to be open on a daily basis.”*

Many participants expressed the need to provide space for children to exercise and play. Several participants suggested promoting rotating “clean air nights” at local businesses to encourage community gatherings and economic stimulation. Some participants discussed the need for a new building. Most participants described the importance of equitable access.

### Community response capacity

Community-level coordination was discussed among participants. Many recommended coordinating resource provision and neighbor-to-neighbor mutual aid. The need to shift away from dependence on external support organizations such as Red Cross was discussed in focus groups: as one participant expressed, *“[we need to] provide food from local sources so the Red Cross doesn’t have to come here and make the food for us. We could take more control. Their food wasn’t that good anyway.”*

### Social gatherings

Many participants discussed the need to gather to boost morale and mental health and wellbeing. Both groups had suggestions for gatherings including concerts, trips to clean air areas, food, dances, yoga, and meditation. One participant stated, *“I would love to see more community events and start to get people out of the house. Get people moving their bodies. See people laughing and smiling again because it’s the morale part of it is probably what is so challenging.”* However, another said that gatherings are difficult to organize during WFS events due to residents leaving the MV, and another expressed that stratified socioeconomic demographics make planning community-wide events challenging.

### Education

Enhanced community-level education on various aspects of WFS was also suggested. Suggested topics included: using air quality apps and understanding AQI; the impacts of WFS events on physical health, mental health, and self-care; approaching WFS events with children; getting clean air in one’s home; mitigating health effects; and mask fitting and use.

### Communications

Suggestions to increase and/or coordinate communication included: implementing informational campaigns during WFS events (including available resources, current and forecasted air quality, burn bans, lowering stress, community events, and which spaces in the community have clean air), expanding information about the interpretation of the Air Quality Index (AQI) (including increasing physical signage throughout the region), providing communications in multiple formats (print, digital, social media, radio, roadside signs, bulletin boards in well-trafficked indoor spaces), and addition of push notifications on local apps when AQI reached harmful levels.

### Volunteerism

Several participants discussed volunteerism to mitigate mental health and wellbeing challenges while helping the community. Volunteer activities that do not center around processing stress, but that allow conversations to arise were emphasized as an opportunity to promote individual-level wellbeing. For example, one key informant stated: *“One of the ways people are strengthened is by doing something, not just standing by, not feeling hopeless, not feeling helpless. And if there could be a work group, some type of organization where people were doing a practical service, not processing verbally necessarily, but getting involved on clearing of a burn pile or joining together with one of the mission groups that came in to help rebuild, I think a lot more people would benefit from doing a practical chore for their neighbors that has been delineated, and they would be much more likely to open up if they were physically involved.”*

### Other

Additional topics discussed by participants included advocacy campaigns for air filter distribution, burn ban communications, and taxes for a clean air community space.

### Toolkit

Interview and focus group findings were used to develop a pamphlet describing WFS wellbeing impacts, evidence-informed strategies to reduce WFS exposure, and WFS event coping strategies suggested by the communities. Interview participants also provided suggestions for content, locations for distribution, medium, and messengers (Fig. 1). Based on their feedback, the pamphlet was printed for distribution in the region, and resources were hosted on Clean Air Methow’s website. The pamphlet was reviewed by interview participants prior to printing and web adaptation. The University of Washington School of Public Health's communications team adapted the pamphlet for distribution to communities outside of the MV in an Instagram story (Additional File 3) during a major regional WFS event in September 2020. The story received over 10,000 likes, 10,800 shares (which had their own likes, shares, and saves), nearly 4000 saves and 50 comments, demonstrating the broad need for such resources during WFS events.

## Discussion

WFS events present physical health and mental health and wellbeing challenges. While physical health impacts have been well described in the scientific literature [2, 23], our findings suggest that impacts to mental health and wellbeing, as well as social and economic conditions foundational to health, are also ubiquitously experienced. In addition to the direct, negative impacts of WFS events on mental health [24], the indirect effects of these events are important to consider as well. These include increased anxiety and worry about the ways in which climate change is associated with an increased number of WFS and the fear of future events [25]. Additionally, affected populations may suffer from the repercussions that are tied to the decreased nature contact that is associated with WFS, through a deprivation of the opportunity to experience the anxiety and rumination reduction [26–28], social cohesion [29, 30], and increases in positive affect [31–33] that nature contact provides.

While few climate change and health vulnerability and adaptation assessments focus on mental health, global literature on mental health and climate change suggests that providing opportunities for individuals to take action against climate risks may support more positive psychosocial responses to climate change [24, 25]. For instance, engagement in groups that incorporate mental health and community-based resilience with activism have been shown to improve overall mental and social wellbeing [34]. Thus, additional research is needed on the topic of WFS events’ effects on mental health and wellbeing, as well as positive and community-based coping mechanisms for people experiencing WFS events.

Our findings suggest that additional resources at the individual and community level are also necessary to mitigate the mental health and wellbeing impacts of WFS events. The number of available care providers in rural communities, particularly those who accept Medicaid insurance, may be inadequate to address the mental wellbeing challenges before, during, and after WFS events. Providing community members with opportunities to learn psychological first aid [35], as well as wellbeing practices such as yoga and meditation, may increase opportunities to support wellbeing in rural communities impacted by WFS. Additionally, exploring options for telemedicine-based therapy that is covered by insurance, as well as integration of mental health into primary care [12], may help expand mental health resources in impacted rural communities.

The importance of social capital in community resilience to disasters has been well described. Social capital networks provide a variety of resources in the context of disasters, including psychosocial support, financial aid, and information [36]. Not surprisingly, our participants also stressed the importance of building community response capacity, and suggested opportunities for “mutual aid,” or for neighbors to help neighbors. Examples included mapping and sharing resources, streamlining communication, and coordinating responses to urgent events, particularly for more at-risk community members. Additional resources should be dedicated to strengthening social connections in rural communities, to enhance ability to adapt and cope to both wildfire smoke and other chronic and acute hazards. Moreover, future research should explicitly explore the role of social capital in enhancing wildfire smoke resilience.

Participants also requested an indoor, temperature-controlled, clean air space where they could seek respite from the smoke, gather with their fellow community members, and engage in the activities they are otherwise unable to do during WFS events (e.g., exercise, children’s activities). Such a space could also provide year-round gathering opportunities during other harsh weather events. Building a new space or adapting an existing space will be an expensive and time-intensive endeavor that will require community input to ensure its utility. Interim solutions proposed by residents included providing or upgrading HVAC systems for existing schools and community centers and opening them to the public during WFS events. Presently, such spaces are limited. Future research should assess the influence of community gatherings and access to community clean air spaces on mental health and wellbeing during WFS events.

Notably, key informants and focus group participants had differing perspectives about the quality and quantity of public health communications during WFS events. Key informants had more positive perceptions about the adequacy and coordination of communications compared to focus group participants. There are several possible explanations for this, including that local health and social service providers received information in the context of their professional role that is not as accessible to the general public, are more aware of information sources and ways to access it, or have disparate levels of wildfire smoke literacy based on their professional training or expertise. As such, additional exploration regarding the reasons for this disconnect is necessary. In the short term, additional resources to facilitate community-level access to WFS information may be needed beyond those anticipated by local health and social service providers.

### Limitations

While focusing this qualitative study in a single, rural community allowed for rich contextual exploration, study findings may not be generalizable to other communities. Moreover, our study included only a relatively small convenience sample of community members who participated in focus groups. Although key informants included practitioners speaking of their in-depth experience with more diverse and at-risk communities, this does not replace self-representation among individuals from these communities. Additionally, due to limited WFS in the MV during summer 2019, smoke events were not at the top of study participants’ minds. Performing research during or just after a WFS event may yield higher participation and deeper reflections.

## Conclusions

As wildfire smoke (WFS) events increase in frequency and magnitude across the Western US and around the world, they continue to pose formidable challenges to physical, mental, and social wellbeing. Community-led solutions that promote stress reduction, physical protection, and community cohesion can increase resilience in the face of these events. Specifically, communities prone to WFS may consider (1 creating a clean air community space, (2) streamlining communications about physical and mental health and wellbeing, and (3) establishing more coordinated, community-driven responses to WFS events.

## Supplementary Information

**Additional file 1.** Focus Group Facilitator Guide.

**Additional file 2.** Key Informant Interview Guide.

**Additional file 3. **Wildfire Smoke and Health Instragram Story Developed by the University of Washington School of Public Health's Communications Team.

## Data Availability

The datasets generated and/or analyzed during the current study are not publicly available due to concerns of privacy and confidentiality but are available from the corresponding author on reasonable request.

## Abbreviations

AQI: Air Quality Impact
MV: Methow Valley, Washington, United States of America
PM: Particulate Matter
WFS: Wildfire smoke
