# Supplementary material for: What can we do when the smoke rolls in? An exploratory qualitative analysis of the impacts of rural wildfire smoke on mental health and wellbeing, and opportunities for adaptation
Source: BMC Public Health. 2022 Jan 6;22:41. doi: 10.1186/s12889-021-12411-2 (PMC8740038; doi:10.1186/s12889-021-12411-2)
Supplement: Supplementary file 1 — Additional file 1. Focus Group Facilitator Guide. [file 12889_2021_12411_MOESM1_ESM.docx]

Additional File 1. Focus Group Facilitator Guide

**Smoke Events Focus Group**

**Facilitator Guide**

*Room setup: Facilitator has a large sticky pad, wellbeing diagram (either printed for each person or drawn on sticky sheet), and 3 sticky sheets hung on opposite wall that say “Individual,” “Community,” and “Town/County”*

*Post-it notes, pens, paper*

1. **Welcome (6:05-6:06)**

Thank you for agreeing to participate in our focus group. My name is [*your name*] and I’m [*your job*]. I am working with Clean Air Methow and a team from the University of Washington to learn about how people in this community are affected by extreme, persistent wildfire smoke events, such as what this community experienced in 2017 and 2018, and how people respond, or could respond to cope with these events. We’re going to use what we learn to design a toolkit that provides community leaders and members with actionable strategies to improve wellbeing during smoke events.

1. **Explanation of the process (6:06-6:08)**

We are using a focus group format to help understand the context behind the answers you will provide to our questions, and to explore topics in more detail than in a survey. We are seeking to learn from you: there are no wrong answers. We’re not trying to get everyone to agree on anything; we’re simply gathering information.

The focus group will last ninety minutes. Help yourself to the refreshments.

We will be taking notes and recording the focus group so that we can refer to the discussion later.

We may write up our findings in a report or for publication in a peer-reviewed journal. We will not refer to you by name in any report or publication.

You can decline to answer any question, and you can leave the focus group at any time.  You will not be penalized for not answering any question or for leaving the focus group.

1. **Ground Rules  (6:08-6:10)**

We do have a few ground rules, based on the norms we’ve got here *gesture to norms*. Everyone should have the opportunity to participate, so if you tend to be a talker, think about allowing space for others to speak. If you tend to be on the quiet side, think about sharing more than you might normally. Information provided in the focus group should be kept confidential. Please do not share what was said or who was here, and respect others’ experiences. Please don’t have side conversations. Turn off cell phones if possible.

We want for you to include the voices of people who you live with who are not present tonight, like your children or elderly parents. However, we don’t want answers for anyone that you didn’t live with during smoke events. So, when you’re answering questions, please answer them only for yourself and the people in your household.

Extended smoke events can be hard or even traumatic for some people and talking about it might bring back some of those feelings. While we honor those emotions, this setting is for information gathering and not a support group. We have a list of local mental health resources available to anyone who wants them. If you start to feel uncomfortably emotional or upset, you are welcome to take some time away to take care of yourself and come back whenever you are ready.

We were lucky to have a relatively smoke-free summer this year. when responding, we would like you to recall the summers of 2018 and 2017, when the Methow experienced several consecutive weeks of poor air quality due to wildfire smoke. In 2018, much of the valley went through 40 consecutive days of unhealthy to hazardous air quality, and the smoke rarely lifted for more than a few hours at a time. While we don’t know for certain what the future will bring, but wildfires and smoke are likely to increase in severity and duration for many years ahead.

Last, we’ve got a lot to cover tonight, and we’re going to stick to a pretty tight timeline. Each of you has a sheet of paper and a pen in front of you. If you feel like you didn’t have enough time to answer a question, or if it’s something you didn’t feel comfortable sharing with the group, please write your response on the paper so that your thoughts are still heard.

1. **Questions and consent (6:10-6:13)**

Does anyone have any questions before we begin?

*Turn on Tape Recorder*

Do you consent to participate in our project? [*Ask everyone to provide a verbal “yes.”*]

1. **Introductions (6:13-6:20)**

Before we begin, I’d like to go around the room and let everyone introduce yourself. Can you please share your first name, the ages of all members of your household the town you live in how long you’ve lived there, and a few words about how you and your household have been affected by smoke events?

1. **Discussion (6:20-6:21)**

Now that we all know each other, I’m going to explain a little bit more about what we are researching. *(pass out the wellbeing diagram)* This is the definition of “wellbeing” we’re using in our research. It’s a balance of physical, psychological, and social resources and challenges. To be in a state of wellbeing, we want to have a balance between resources and challenges on all three dimensions. We’ve used this diagram to guide the questions I’ll be asking you over the next hour or so. Does that make sense? *If not, clarify whatever is confusing; once they are on the same page:*

***Challenges (6:20-6:40)***

Great; we can dive right in to our questions. I’m going to ask you questions about wildfire smoke, and I ask you to think back to a specific time or times when you and the people in your household with had to live with heavy wildfire smoke for an extended period. We’re going to start with challenges. I’m going to summarize some of what you say on this big sheet of paper. We’ve got about 20 minutes for this part of the discussion. Please think about your experience, along with the experience of members of your household. *About five minutes for each question*

1. How did wildfire smoke impact you physically?

1a. Were you able to do the physical activities you normally do?

1. How did wildfire smoke impact your social life?
2. How did wildfire smoke make you feel?
3. Did wildfire smoke impact your ability to do things you enjoy, and if so, how?

*From what you wrote, form a sentence to summarize and ask if it was accurate. If not, rephrase until you get it right.*

***Wellbeing***

Thank you everyone. Now we’re going to shift gears and talk about resources and opportunities for being better able to cope with wildfire smoke. We’ll start with an activity, and then have a conversation.

6:40-6:57

I’m going to give each of you Post-it notes. You can use as many of them as you like to write down resources or services you can think of, either ones that already exist or what you would like to see to improve wellbeing during wildfire smoke events. You’ll stick each Post-it onto the big sheet of paper that it fits into, category-wise. The categories are: individual (things you can do for or by yourself or with your household), community (things you can do with your community or things your community can do with you), and town/county (things you can do with your town, or things your town or providers can do for you). Put the things that are already available to you on fushia stickies, and things you’d *like* to see in aqua. Make sense? *Answer any questions*

Great! *Pass out sticky notes and pens* I’m going to put seven minutes on the clock. Come up with as many answers as you can. I’ll be observing and arranging the Post-its around when I see similarities between them. Then we’ll regroup and talk about what we see!

*Reveal “Activity Prompt” large sticky notes. If anyone seems stuck, you can refer them to the posted prompts post:*

- Was there anything you did that helped when the air was so bad you didn’t want to leave your house and go outside? How did you pass the time?
- Did any of your care providers, like a doctor or counselor, give you any good advice?
- Did your town/county government offer any kind of help?

*Watch what people are posting, and, without getting in the way, rearrange them into categories that fit together (eg. Community/clean air space, Indoor exercise, Support groups—whatever you see). Start to think about commonalities you’re seeing.*

Okay, thank you all so much.

6:57-7:20

*For the answer with the most fuschia stickies:*

It looks like [resource] is something that a lot of people found helpful.

How has it impacted your wellbeing? [Prompts: your physical wellbeing? your social wellbeing? your mental wellbeing?]

How did you learn about it? How could we make it more widely available?

*For the resource with the most aqua stickies:*

I’m seeing a lot of interest in [resource], but the aqua stickies tell me that that hasn’t been offered around here. What would a [resource] look like?

Where would it be?

How could it impact your wellbeing? [Prompts: your physical wellbeing? your social wellbeing? your mental wellbeing?]

*For the answer with the most divided aqua-fuschia*

There were a lot of mentions about [resource], and it seems like it’s a mixed bag—about half of you indicated that it was available to you, and about half said it wasn’t. For those of you who said it was available, how did you access it? How did it impact your wellbeing? [Prompts: your physical wellbeing? your social wellbeing? your mental wellbeing?]

For those who said it wasn’t available, what would you want to see?

*Once you’ve covered the largest categories:*

7:20-7:30

If we had to prioritize two or three, what would they be?

- Why?
- What role can your town or service providers play in implementing these interventions?

7:30-7:37

Thank you all so much; all of these ideas are great! I’ve just got one more question before we wrap up, and it’s a sort of pie-in-the-sky question: What would make you the happiest or healthiest during a smoke event? Let’s go around the circle for this one.

7:37-7:40

1. **Closing**

Thank you very much for your time. This has been incredibly informative.

- Is there anything else you’d like to tell us about wellbeing, smoke events and your community? You’re also welcome to write down any other thoughts.
- Does anyone have any questions?

You can contact Anna at any time if you have additional input, questions or concerns about this topic or project.  *Provide participants with business cards.*
