# Supplementary material for: What can we do when the smoke rolls in? An exploratory qualitative analysis of the impacts of rural wildfire smoke on mental health and wellbeing, and opportunities for adaptation
Source: BMC Public Health. 2022 Jan 6;22:41. doi: 10.1186/s12889-021-12411-2 (PMC8740038; doi:10.1186/s12889-021-12411-2)
Supplement: Supplementary file 2 — Additional file 2. Key Informant Interview Guide. [file 12889_2021_12411_MOESM2_ESM.docx]

Additional File 2: Key Informant Interview Guide

**Wildfire Smoke Event Wellbeing Toolkit**

**Key Informant Interview Guide**

**Researchers’ Statement**

I am asking you to participate in an interview as a key informant. I will now provide you with the information you will need to help you decide whether to participate. You may ask questions about the purpose of the project, what I would ask you to do, the possible risks and benefits, your rights as a participant and anything else about the project that is not clear. When I have answered all your questions, you can decide if you want to participate or not. This process is called “informed consent.”

**Purpose of the Study**

I am interested in learning from you about how persistent, extreme wildfire smoke events affect the wellbeing of your community, and how individuals and community organizations are responding, or could respond to, these impacts. For the purposes of this project, wellbeing is defined as a balance between physical, psychological, and social resources and challenges. I wanted to speak with you because of your connection to community members in the Methow Valley. As an incentive, I am offering participants the option of receiving a copy of Brian Fies’ A Fire Story, a graphic novel that describes his experience following the Tubbs wildfires.

**Study Procedures**

My project team and I have conducted focus groups with Methow Valley residents, and are now conducting interviews with health and social service providers in the community. Each interview will last 30 to 45 minutes. I will not ask sensitive or personal questions during this interview; my questions focus on your professional experience and perspectives regarding your community’s experience during extreme, persistent wildfire smoke events, and resources you know to be available or would like to see that can enhance wellbeing during wildfire smoke events.

**Risks, Stress, or Discomfort**

Some of these questions might cause you discomfort as you think about the overwhelming nature of extreme smoke events. You can refuse to answer any question or discontinue the interview at any time. There are no repercussions for refusing to answer any question or for discontinuing the interview, and you can still elect to receive the study incentive, a copy of Brian Fies’ A Fire Story.

**Benefits to the Study**

This interview might help you think more broadly about how to help your community respond to smoke events. As a whole, the findings from our project will identify valuable information about how the Methow Valley community is responding to the wellbeing impacts of extreme, persistent smoke events, as well as opportunities to improve wellbeing and resilience.

**Confidentiality of Research Information**

I would like to audio record this interview to ensure I am accurately summarizing your responses in our final reports. I will send this recording to a transcription service to transcribe the interview.

Outputs of this project will include a toolkit that outlines strategies to protect and improve wellbeing during wildfire smoke events and a written report describing our findings. The report will only include aggregated data, and my team and I will not include your name or the names of other interviewees in the report. Despite these steps to protect your identity, it may be possible to identify you based on your responses or other details that you share with us today.

Questions

Do you have any questions?

Do you consent to participate in this interview?

Do you consent to have this interview recorded?

Can you please describe your role in the community as a service provider?

How long have you been in your current role?

What has been your professional role related to wildfire smoke? [Prompts; Before an event, after an event]

Can you please describe the population your organization provides services for?

How have you seen the population you serve affected by wildfire smoke events?

Physically?

Socially?

Psychologically?

How has the population you’ve been working with responded to wildfire smoke events?

Physically?

Socially?

Psychologically?

As I mentioned earlier, we are going to build a toolkit for wellbeing resilience during wildfire smoke events, which will be available to all Methow Valley community members, leaders, and health and social service providers. This will be available online and through community care providers. Now we are going to talk about resources that are available to the community or what you’d like to see. We will build your responses into the toolkit.

What do you see as opportunities to address wellbeing impacts during wildfire smoke events experienced by your clients or community?

Physical?

Social?

Psychological?

What could your organization do to help?

How could the toolkit be disseminated in the community you serve?

What medium would be best-- physical pamphlets, website, something else?

Who would be the best messenger for this information?

Is there anything else you’d like to tell us?

Is there anyone else that is in this field who you think we should also talk to?

We’re offering a copy of A Fire Story, a graphic novel by Brian Fies on his experience following the Tubbs Wildfires, to interviewees. Would you like a copy of the book? And if so, what address can we send it to?
