# Supplementary material for: What can we do when the smoke rolls in? An exploratory qualitative analysis of the impacts of rural wildfire smoke on mental health and wellbeing, and opportunities for adaptation
Source: BMC Public Health. 2022 Jan 6;22:41. doi: 10.1186/s12889-021-12411-2 (PMC8740038; doi:10.1186/s12889-021-12411-2)
Supplement: Supplementary file 3 — Additional file 3. Wildfire Smoke and Health Instragram Story Developed by the University of Washington School of Public Health's Communications Team. [file 12889_2021_12411_MOESM3_ESM.pdf]

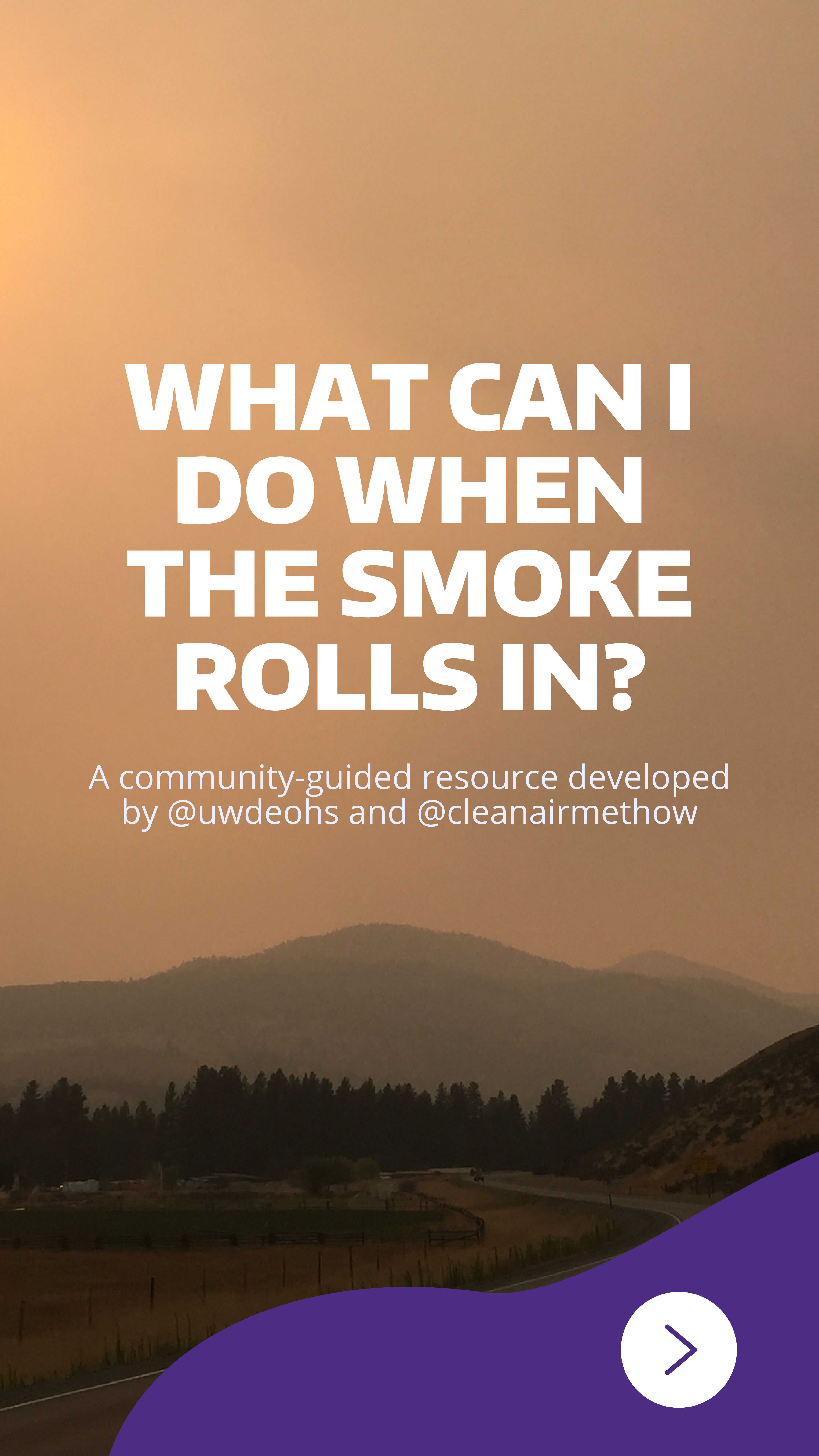
WHAT CAN I DO WHEN THE SMOKE ROLLS IN?
A community-guided resource developed by @uwdeohs and @cleanairmethow

PROTECT YOUR BODY

Smoke can get caught deep in your lungs and enter your bloodstream. This can cause problems now or later.

Some symptoms are common.
During smoke events, lots of people have irritated eyes, a cough, headaches, sleeplessness, or slight shortness of breath. However, you should contact your doctor or call 911 if you have trouble breathing or chest pain.

Some people are higher risk.
If you have asthma or another respiratory disease, heart disease, you're over 64, or you smoke cigarettes, you're at higher risk for serious complications. Babies, children, and pregnant women also need to be extra careful.

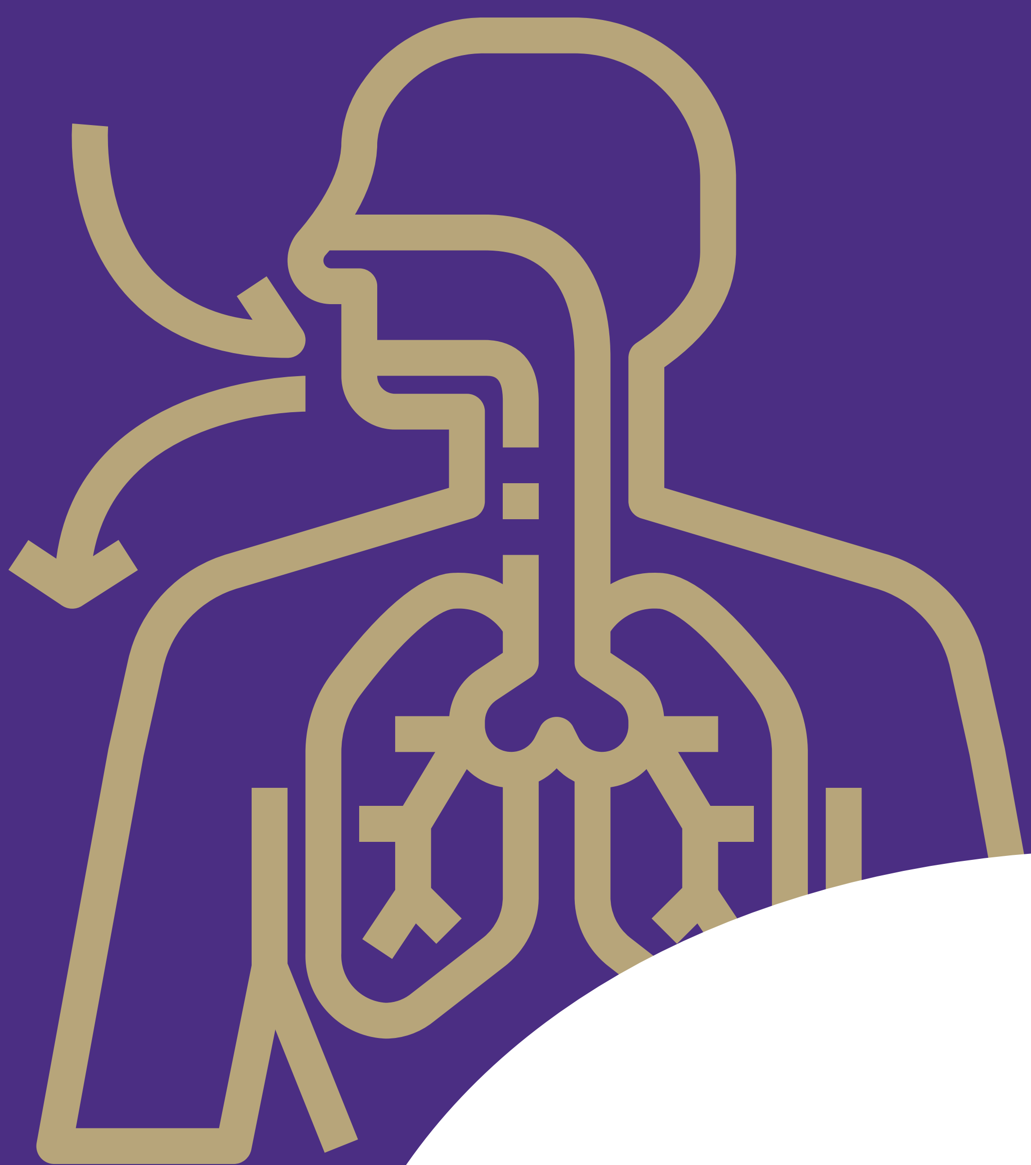

REDUCE SMOKE INDOORS:

- If you're lucky enough to have it, use your AC set to re-circulate (this may not be possible in all systems. Check yours - the goal is to reduce pulling in outside air). If that's out of your price range, buy an air filter or learn to build your own by attaching a MERV 13 filter or higher to a box fan. Video here: tinyurl.com/boxfilter.

- Stay inside with doors and windows closed.

- Don't burn candles, use gas stoves, smoke tobacco products, vacuum, or fry food.

- If you don't have an AC and it's too warm to stay inside, go to a friend's house or a community space or even the grocery store just for a "breather."

- It's important to continue to follow COVID-19 recommendations for public spaces, including physical distancing and wearing a mask. There might be reduced capacity at some facilities.

- Pay attention to when air quality improves during smoke events, often in the evenings during the summer. Use this chance to open your windows to cool and air out your home.

REDUCE EXPOSURE OUTSIDE:

- Cloth masks generally do not provide a lot of protection against wildfire smoke. N95 respirators can provide protection if worn properly, but need to be saved for health care and frontline workers while supplies are limited and we are fighting COVID-19.

- Turn the AC on recirculate mode in your car.

- Avoid or reduce activities that make you breathe hard or deeply. See if you can exercise indoors. Check the air quality on the Methow Air app (for those in the Methow Valley), airnow.gov, or with the Forest Service.

- Try to save yourself some errands. Have enough food and medication for a few days.

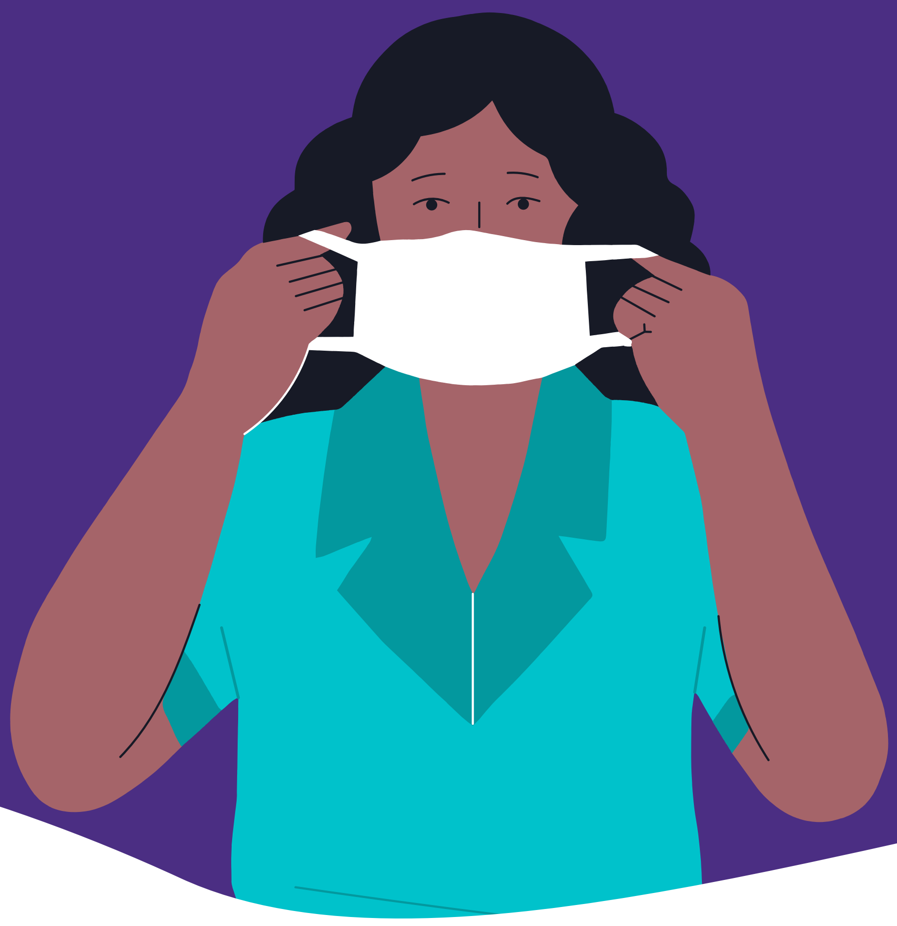

ABOUT THOSE FEELINGS...

It's okay to get in a funk.

Long wildfire smoke events can really suck. Lots of people feel sad, anxious, dull, hopeless, or irritable, and these emotions can last after the smoke is gone. Your appetite or sleeping habits might change, and you might have low energy. It's normal, but there also are things you can do to feel better.

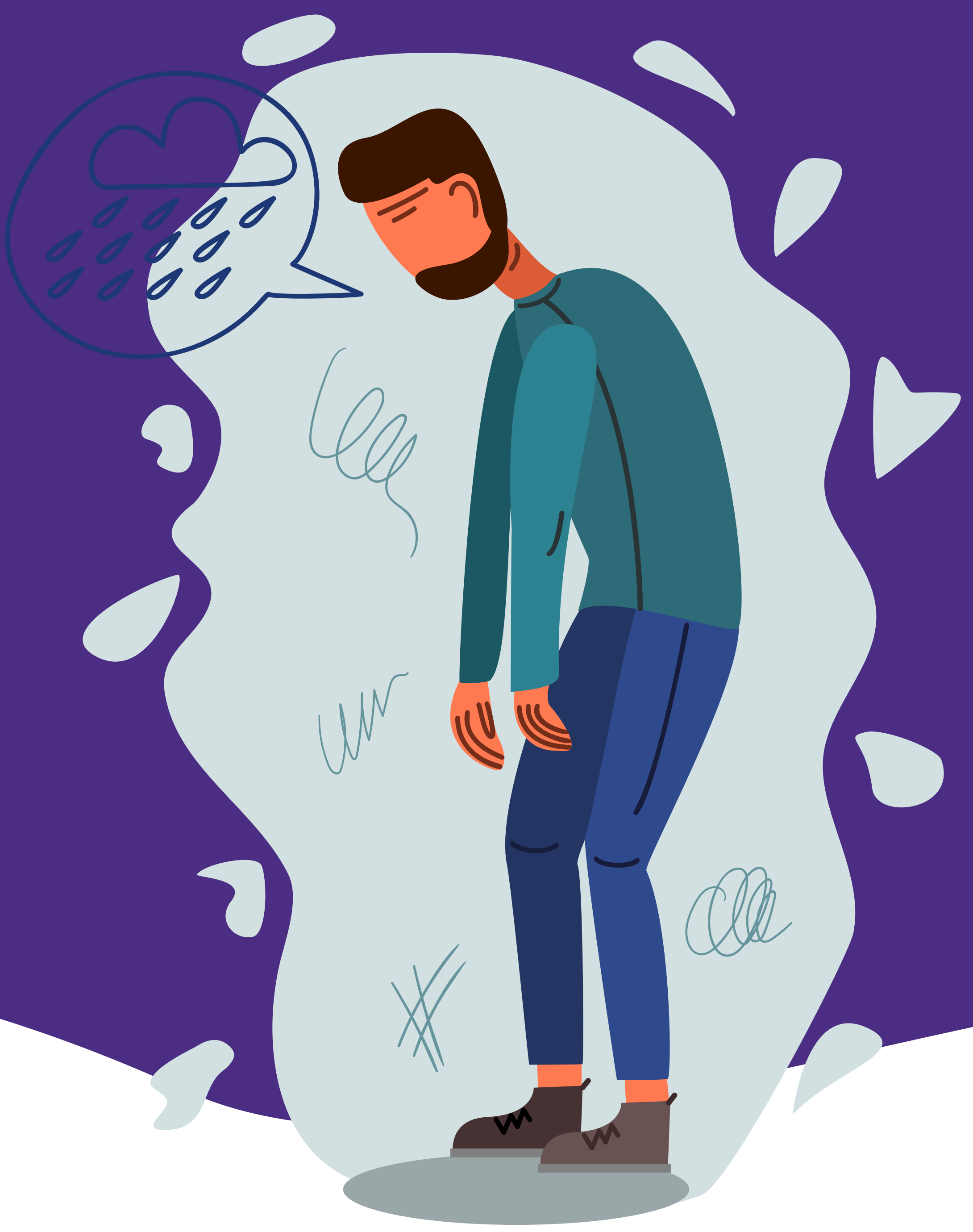

- **Call your friends and family**
  Bonus points if you talk about something besides the smoke!

- **Get some light exercise**
  Doing light weights, dancing, stretching or yoga, or walking around your house can be good for the mind and body. Just don't do anything that makes you breathe too hard if your indoor air is smoky!

- **Find a hobby**
  Pick up that dusty guitar, colored pencils, or knitting needles.

- **Seek professional help or a support group**
  Sometimes asking for help is the strongest thing you can do.

- **Take time to breathe**
  An easy start is to sit comfortably, close your eyes or keep them half open. Breathe in through your nose for three counts, and out through your nose for four counts. When your mind wanders away with you, just come back to paying attention to your breath. Even a few minutes of breathing like this can help calm the nervous system.

- **Listen to music that makes you feel good**

- **Lower the bar**
  Expecting to have a great day while stuck inside may be setting yourself up for failure. Try to find a few small moments of appreciation or happiness, but give yourself a break.

WE'RE IN THIS TOGETHER

Smoke events can be a time to connect with each other, ask for what you need, and help others.

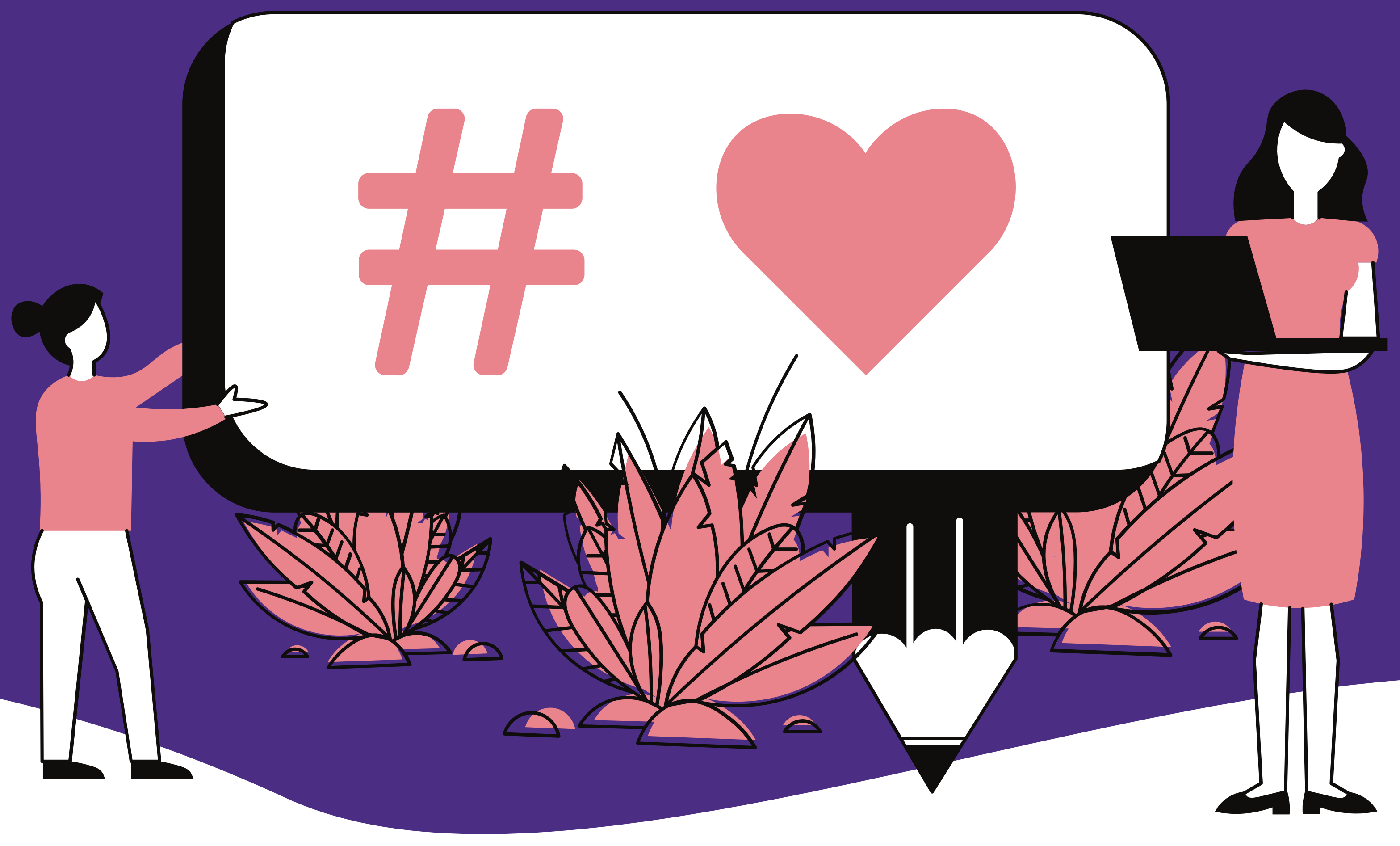

- **Check in on your friends and neighbors**
  This includes people you don't really know! Can you set up a phone tree? Run an errand for someone who can't leave their house? Try to have regular contact.

- **Make a resource map**
  Ask your neighbors what resources they have and are willing to offer to others, such as a cool basement, an extra air purifier, ability to run errands for others, etc. and keep it on a map.

- **Find ways you can get together**
  Seek out a space with clean(er) air where you could have a potluck, game night, dance party, or get kids together to play. But, continue to follow physical distancing measures and COVID-19 considerations in your county.

- **Find a place to volunteer**

- **Create a clean air community space**
  Smoke events probably won't get better any time soon. More than anything else, your community asked for a space to exercise, hang out, and be together. If you want to prepare in a big way, voice your support for a space like this with your neighbors.

  Follow the CDC's guidelines for community air spaces during COVID-19.
